# Biochar Amendments and Phytoremediation: A Combined Approach for Effective Lead Removal in Shooting Range Soils

**DOI:** 10.3390/toxics12070520

**Published:** 2024-07-19

**Authors:** Rocio Maceiras, Leticia Perez-Rial, Victor Alfonsin, Jorge Feijoo, Ignacio Lopez

**Affiliations:** Defense University Center, Spanish Naval Academy, Plaza de España s/n, 36920 Marín, Spain; leticia@cud.uvigo.es (L.P.-R.); valfonsin@cud.uvigo.es (V.A.); jfeijoo@cud.uvigo.es (J.F.); ignaciolopezhuertas@gmail.com (I.L.)

**Keywords:** biochar, lead-contaminated soils, shooting range, seed germination, *Brassica rapa*, *Lolium perenne*

## Abstract

The increasing contamination of soil with heavy metals poses a problem to environmental sustainability. Among these pollutants, lead is particularly concerning due to its persistence in the environment, with harmful effects on human health and ecosystems. Various strategies that combine phytoremediation techniques with soil amendments have emerged to mitigate lead contamination. In this context, biochar has gained significant attention for its potential to enhance soil quality and remediate metal-contaminated environments. This study aims to investigate the combined effect of biochar amendments on the phytoremediation of lead-contaminated shooting range soils. A series of experiments were conducted to determine the impact of the amount and distribution of biochar on lead removal from soil. Soil samples were incubated with biochar for one week, after which two types of seeds (*Brassica rapa* and *Lolium perenne*) were planted. Plant and root lengths, as well as the number of germinated seeds, were measured, and a statistical analysis was conducted to determine the influence of the amendments. After one month, the Pb concentration decreased by more than 70%. Our results demonstrate that seed germination and plant growth were significantly better in soil samples where biochar was mixed rather than applied superficially, with the optimal performance observed at a 10% wt. biochar amendment. Additionally, the combined use of biochar and phytoremediation proved highly effective in immobilizing lead and reducing its bioavailability. These findings suggest that the combination of biochar, particularly when mixed at appropriate concentrations, and *Brassica rapa* significantly improved lead removal efficiency.

## 1. Introduction

The contamination of soil with heavy metals, such as lead (Pb), poses significant environmental and health risks worldwide. Lead, a toxic metal commonly found in industrial areas, mining sites, and urban soils, can persist in the environment for extended periods, causing adverse effects on ecosystems and human health [1]. Lead at shooting ranges is a significant environmental concern due to the amount of lead waste generated and accumulated at these sites due to its widespread use in ammunition. Bullets are primarily composed of lead because of its high density, which ensures effective energy transfer upon impact, and its relatively low cost [1]. Lead azide is used in primers to activate a bullet when struck by the firing pin. This causes lead particles to be released into the air as dust when the firearm is discharged, contributing further to environmental contamination. The repeated discharge of firearms in these shooting ranges results in the accumulation of lead particles in the soil and dust, making the contamination substantial [2]. For that reason, both commercial and private shooting ranges are considered major sources of environmental lead contamination [3]. According to Mendes et al. [4], activity at shooting ranges contributes millions of pounds of lead to the environment annually, surpassing most industries except for metal mining and manufacturing. This lead not only remains on site but can also leach into water sources and affect nearby wildlife and properties. For that reason, it is necessary to look for adequate management and mitigation strategies at shooting ranges to reduce lead exposure and protect both human health and the environment.

Traditional remediation methods for Pb-contaminated soils [5,6,7] (electrokinetic remediation, soil excavation, soil washing, thermal desorption, chemical stabilization) often involve high costs and may have limited effectiveness in achieving long-term soil restoration [8,9]. For that reason, different approaches combining phytoremediation techniques with soil amendments have emerged as promising strategies for sustainable soil remediation [10,11].

Among the various soil amendments, biochar has garnered considerable attention due to its unique physicochemical properties and potential to improve soil quality and enhance plant growth [12]. Biochar, a carbonaceous material produced from the pyrolysis of organic biomass, offers numerous benefits, including increased soil porosity, enhanced nutrient retention, and reduced bioavailability of contaminants [13]. When integrated with phytoremediation, which utilizes plants to extract, degrade, or immobilize contaminants from soil and water [11], biochar has been shown to augment the efficiency of metal uptake by plants, reduce metal leaching, and improve soil conditions, thereby facilitating the remediation of Pb-contaminated soils [14]. Moreover, biochar can increase the uptake and sequestration of Pb by plants, accelerating the remediation process [15]. Numerous studies have investigated its efficacy in diverse soil types, climatic conditions, and contaminant scenarios [16,17]. Research findings have consistently demonstrated the ability of biochar to enhance soil structure, water retention, nutrient availability, and microbial activity, thereby promoting plant growth and productivity [18]. Moreover, biochar-mediated changes in soil’s physicochemical properties have been shown to influence the bioavailability and mobility of various contaminants, including heavy metals, organic pollutants, and excess nutrients, leading to their sequestration, degradation, or immobilization within the soil matrix [15,19,20].

Phytoremediation studies have studied the efficacy of *Brassica rapa* and *Lolium perenne*. *Brassica* species have been proven to be effective in the phytoextraction of various heavy metals like Pb, Cr, Cu, Cd, Ni, and Zn, utilizing defense mechanisms such as antioxidant enzymes and amino acids to alleviate metal stress without compromising plant growth and development [21]. Asikin et al. [22] studied the heavy metal tolerance of *Brassica* species and found that *B. rapa* species possess strong tolerance and accumulation capabilities for non-essential heavy metals (Cd, Cr and Pb), making them potential hyperaccumulators for green remediation techniques in toxic soil environments. *Lolium perenne* is capable of absorbing metal into the root matrix, making it a potential candidate for the phytoremediation of landfill soil and the phytostabilization of Cu, Cr and Pb [23]. Li et al. [24] found that *Lolium perenne* decreased the content of lead in soil by 44%.

Despite growing interest in the combined use of biochar and phytoremediation, there remains a need to study the factors influencing their effectiveness in lead remediation. For this reason, the objective of this research is to investigate the combined effect of biochar and phytoremediation (*Brassica rapa* and *Lolium perenne*) on Pb remediation in shooting range soil and to identify the factors that contribute most significantly to reducing Pb content in soil.

## 2. Materials and Methods

### 2.1. Material Collection and Characterization

Soil samples were collected from the “General Morillo” military base in Pontevedra (42°23′9″ N and 8°39′15″ W), which is used as a shooting and maneuver range of the Organic Polyvalent Brigade “Galicia” VII (BRILAT). Samples were collected from tree zones: zone 0, or control (soil without contamination); Zone 1, located one meter from the firing line (named Z1); and Zone 2, located at the end of the shooting range behind the target area (named Z2). All samples were collected at a depth of approximately 20–30 cm.

The samples were dried at 25 °C and manually sieved at 2 mm. pH and conductivity were measured at a ratio of 1:10 (soil/ultrapure water). Pb and S concentrations were measured in aqueous extraction by inductively coupled mass spectrometry (ICP-MS) using an ICP-OES iCAP-pro (ThermoFisher, Karlsruhe, Germany). For this purpose, about 0.2 g of powdered soil samples were solubilized by acid digestion with 3 mL nitric acid, 3 mL hydrogen peroxide and 1 mL hydrochloric acid in Teflon digesters in a SYNTHOS 300 microwave oven (Anton Paar, Graz, Austria). The samples were then diluted to 50 mL with ultrapure water, shaken and filtered for subsequent analysis.

The soil fraction from zones 1 and 2 was divided into aliquots to analyze both the effect of the amount of biochar (5, 10 and 15% wt.) and its distribution (surface, named S, or mixed, named M). Biochar was purchased from LivingChar (Barcelona, Spain). This biochar is produced from wood residues by pyrolysis. This 100% organic product contains a high content of stable organic carbon. Its elevated stable organic matter content and high porosity are valuable properties that make this biochar an ideal material for improving the health and fertility of contaminated soils, as well as for inhibiting soil toxicity from heavy metals and toxic organic compounds. Its characteristics, provided by the supplier, are presented in Table 1. Before mixing with the soil, biochar was manually sieved at 2 mm.

**Figure 1:**
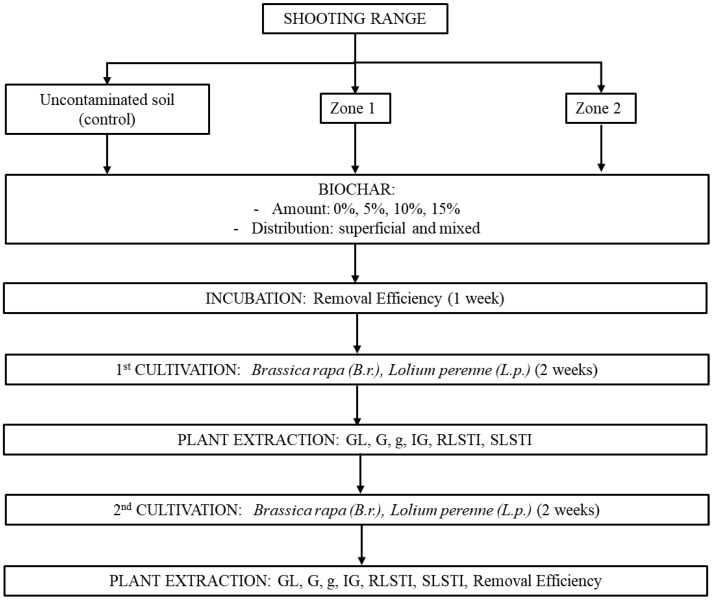
Process scheme.

In all experiments, 500 g of each zone (1 and 2) was used to fill containers (12.5 cm × 19 cm × 4.5 cm) and subjected to an incubation period with biochar and subsequently to phytoremediation in two steps. The same amounts of uncontaminated soil (used as a control and taken from zone 0) and contaminated soil without biochar were subjected to the same process. Each experiment was carried out in duplicate. The scheme of the process is presented in Figure 1, and Table 2 gives the nomenclature of each soil sample.

### 2.2. Material Collection and Characterization

The incubation of amended soils was carried out over one week at 25 °C in closed containers. The containers were prepared with 500 g of contaminated soil and different amounts of biochar (5, 10 and 15% wt.) using two distributions: biochar mixed with soil (distribution 1) and biochar added to the soil surface (distribution 2). Water was added (150 mL) until the soil reached 70% humidity, measured with a moisture analyzer (MA 110.R, Radwag). pH was monitored at a ratio of 1:10 (soil/water) every two days, and Pb content was measured by mass spectrometry (ICP-MS) at the end of the period.

After one week of incubation, seeds of *Brassica rapa* and *Lolium perenne* were planted in each container. The containers were divided into two halves, allocating a half for each crop. The same number of seeds were planted, a total of 12 seeds of each type, distributing them into 3 rows and 4 columns, approximately 2 cm apart vertically and horizontally. The humidity was adjusted every two days by weighing. pH was monitored at a ratio of 1:10 (soil/water) every two days. The plants’ growth after two weeks was measured according to the number of germinated seeds, the length of the plants and the shoot length. *Brassica rapa* necessitates approximately 4 days to achieve initial growth, whereas *Lolium perenne* requires around 7 days to reach a similar developmental stage. A period of two weeks was selected to ensure complete and observable plant development.

After plant extraction, new seeds of *Brassica rapa* and *Lolium perenne* were planted again in the same soil for another two weeks. In both cultures, the containers’ position was changed every two days to avoid any possible influence of microclimatic conditions (25 ± 1 °C). pH was monitored at a ratio of 1:10 (soil/water) every two days, and Pb content was measured by mass spectrometry (ICP-MS) at the end of the period. The plants’ growth after two weeks was measured according to the number of germinated seeds, the length of the plants, and the shoot length.

### 2.3. Data Analysis

The removal efficiency (%) of each sample was calculated using Equation (1) at the end of incubation and cultivation. Moreover, in distribution 2 (biochar added to the soil surface), the biochar fraction and the soil fraction were analyzed separately, using the latter to determine the removal efficiency (%). However, when biochar was mixed with soil (distribution 1), a homogeneous sample was analyzed due to the impossibility of separating soil and biochar.

(1) $$Removal\;efficiency\;\left(\%\right)\;=\;\frac{\left(C_{n}-C_{0}\right)}{C_{0}}\times 100$$

where *C*_0_ and *C_n_* are the metal concentrations at the beginning and the end of the process for each sample, respectively.

The toxicity of the soil after incubation and after the cultures was studied for two species: *Brassica rapa* and *Lolium perenne*. In each container, the germination speed (GS), germination percentage (G), germination coefficient (g), germination index (IG), root length stress tolerance index (RLSTI) and shoot length stress tolerance index (SLSTI) were evaluated according to the following equations [25,26]:

(2) $$GS\left(seeds\cdot {day}^{-1}\right)=\frac{S_{1}}{D_{1}}+\frac{{S_{2}-S}_{1}}{D_{2}}+\cdots +\frac{{S_{n}-S}_{n-1}}{D_{n}}$$

(3) $$G\left(\%\right)=\frac{S_{n}}{S_{0}}\times 100$$

(4) $$g\left(\%\right)=\frac{S_{n}}{S_{c}}\times 100$$

(5) $$IG\left(\%\right)=\left(\frac{G_{n}}{G_{c}}\times \frac{L_{n}}{L_{c}}\right)100$$

(6) $$RLSTI\left(\%\right)=\frac{{RL}_{n}}{RL_{c}}\times 100$$

(7) $$SLSTI\left(\%\right)=\frac{{SL}_{n}}{SL_{c}}\times 100$$

where *S*_0_ is the total number of planted seeds; *S*_1_, *S*_2_ and *S_n_* are the numbers of germinated seeds on the first, second and *n*th day, respectively, for each sample; *D*_1_, *D*_2_ and *D_n_* are the numbers of days after sowing; *S_c_* is the number of seeds germinated in the uncontaminated soil (control); *G_n_* and *G_c_* are the germination percentages in each sample and in the uncontaminated soil (control); *RL_n_* and *RL_c_* are the root lengths of the stressed and control plants, respectively; and *SL_n_* and *SL_c_* are the shoot lengths of the stressed and control plants.

### 2.4. Statistical Analysis

A two-way ANOVA was used to carry out a statistical analysis on the effect of the amount of biochar and its distribution on different soil and plant properties. In the statistical tests, a *p*-value of 0.05 was considered significant, and less than 0.01 was considered very significant. The analyses were conducted using Excel 2016 for Windows.

## 3. Results and Discussion

### 3.1. Soil Characterization

The total Pb concentration in the studied shooting range soils exceeded the normal levels of lead found in unpolluted soils. According to Galician legislation, there are three contamination thresholds for Pb concentrations based on land use: 80 mg/kg for ecosystem protection, 100 mg/kg for urban use and 500 mg/kg for industrial use [27]. Furthermore, the total Pb concentration varied depending on the location within the shooting range, with significantly higher levels in Zone 2 (Z2), measuring 116,000 mg/kg (located behind the target area), compared to 170 mg/kg in Zone 1 (Z1). This disparity highlights the urgent need for soil amendments in these areas. Table 3 summarizes the initial values of different soil parameters.

The pH of the uncontaminated soil exhibits a slightly acidic nature, which is characteristic of Galician soils with low fertility, where nutrients are sequestered by the available aluminum, which binds nutrients and reduces their availability [28]. In contrast, the pH in zones Z1 and Z2 is basic. This shift towards alkalinity may be attributed to the release of heavy metals such as Pb, Cu and Zn into the soil [29]. Heavy metal contamination is known to elevate soil pH, as metals like lead, copper and zinc can neutralize soil acidity through various chemical interactions [30,31]. Additionally, the conductivity measurements of all three soil samples are very low, which indicates that there are hardly any ions dissolved in the medium. This could be due to the soil’s low moisture content or the minimal presence of saline ions and available nutrients [32].

### 3.2. Effects of Amendments on Soil pH

**Figure 2:**
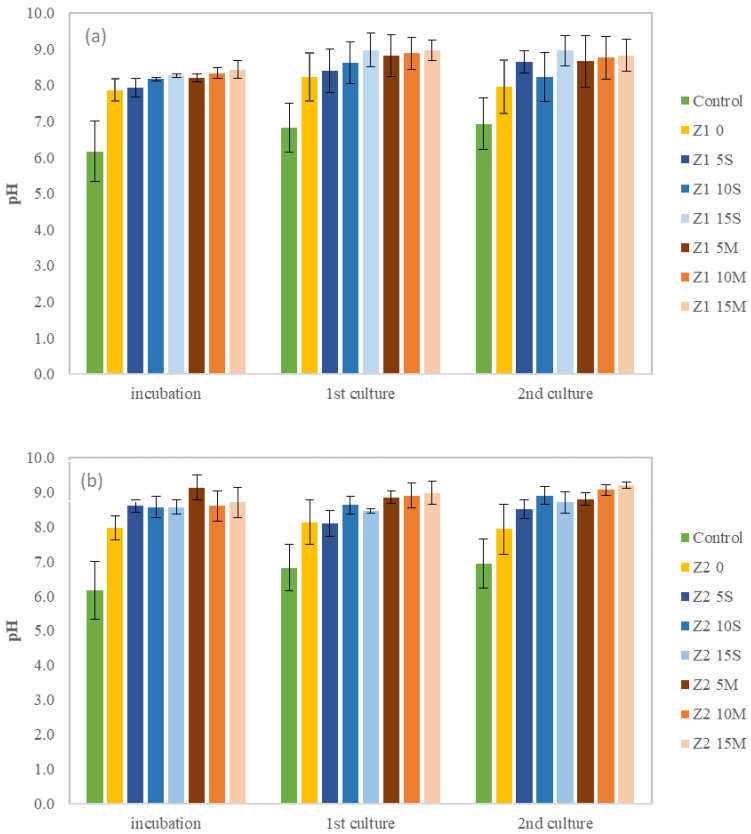
Mean pH variation for the studied soils over the experimental period: (**a**) Zone 1 (Z1) and (**b**) Zone 2 (Z2).

The soil pH was monitored during the incubation and germination periods to evaluate the impact of amendments. The pH of the contaminated soil was approximately 8.0, while the pH of the uncontaminated soil was below 7.0. Figure 2 shows that the addition of biochar increased the soil pH to alkaline levels during the incubation period. Biochar can increase soil pH through several mechanisms that involve its inherent chemical properties and interactions with soil components [33,34]. When biochar is incorporated into the soil, it can release carbonates and bicarbonates. These compounds react with hydrogen ions in the soil, thereby reducing the soil’s acidity and increasing its pH [16]. Moreover, biochar has a high cation exchange capacity, which allows it to retain and exchange essential nutrients in the soil. The presence of biochar can enhance the soil’s ability to hold onto cations such as calcium, magnesium and potassium, which are alkaline and can contribute to raising the soil’s pH [33]. Yuan et al. [35] examined the alkali content in biochar produced from various crop residues at different temperatures, finding that biochar contains significant amounts of alkali metals and alkaline earth metals, which can neutralize soil acidity and thereby increase the soil pH.

After the incubation process, the pH of the contaminated soil remained stable during the first and second cultivation periods in both zones. Furthermore, an increase in the pH of the uncontaminated soil was observed during seed cultivation. These results indicate that this amendment could be particularly suitable for acidic soils [36].

The *Brassica rapa* seeds germinated in a pH range of 7.9 to 9.0, whereas the Lolium perenne seeds did not germinate under these conditions. This discrepancy can be attributed to the differing pH tolerance ranges of the two species. *Brassica rapa* is known to be more tolerant to alkaline conditions, allowing for its successful germination and growth within this pH range [37]. In contrast, *Lolium perenne* typically prefers more neutral-to-slightly acidic conditions for optimal germination [38]. The lack of germination in *Lolium perenne* seeds under alkaline conditions highlights the importance of soil pH in influencing seed germination and species-specific adaptability to different soil environments.

### 3.3. Effects of Amendments on Pb Levels

**Figure 3:**
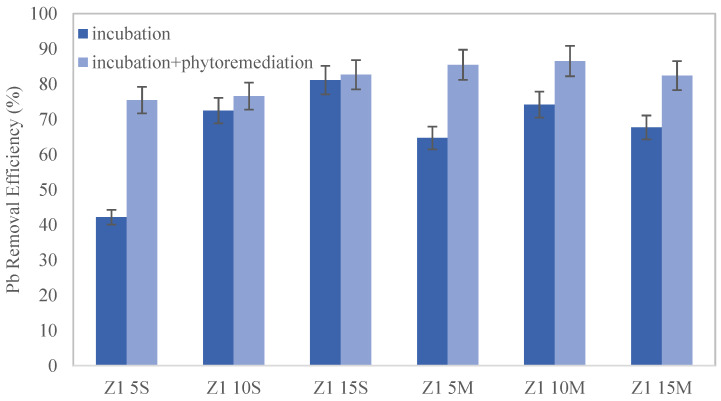
Removal efficiency after amendments in Zone 1.

The lead levels in the shooting range samples exceeded the maximum permissible limits, particularly in Zone 2 (Z2). However, these levels were successfully reduced by more than 70% in both zones. Figure 3 shows that the combined effect of biochar with phytoremediation was highly effective in Zone 1 (Z1), with removal percentages exceeding 75% in all cases. The high percentages of lead removal can be attributed to the substantial accumulation of lead in the biochar. The analyses demonstrated a significant increase in the amount of lead adsorbed by the biochar over the experimental period. Specifically, in Zone 1, the concentration of lead in the biochar increased from 2.7 mg/kg to 20 mg/kg, while in Zone 2, it surged dramatically to 1400 mg/kg. These findings indicate biochar’s exceptional capacity for lead adsorption, consistent with its known high surface area, porosity, and abundance of functional groups that facilitate metal binding [15].

According to the distribution, the best results were obtained when biochar was homogeneously mixed with the soil at 10% wt. biochar (Z1 10M), although the differences between results are not statistically significant. Regarding the incubation period when biochar is distributed on the surface, it is observed that 5% wt. biochar is insufficient to achieve satisfactory recovery, and the percentage increases with the amount of biochar. However, in the case of biochar mixed with the soil, the yields during the incubation period appear lower, which could be due to an accumulation of lead in the plants, although [15] it is most likely due to the dilution of the lead effect when soil is mixed with biochar, making it seem like there is less lead in the soil. On the other hand, there is a maximum effect when 10% wt. biochar is added.

**Figure 4:**
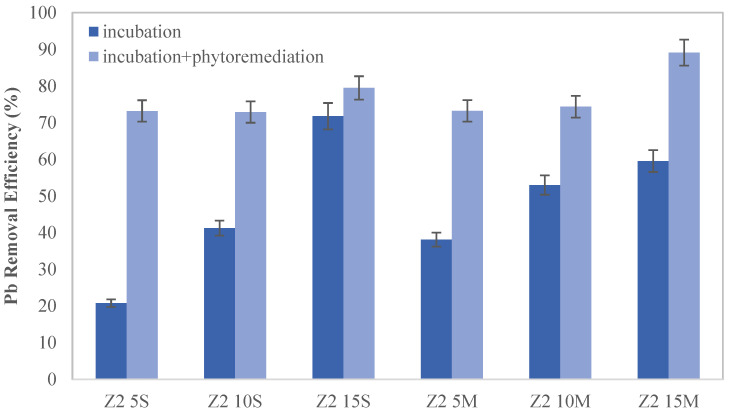
Removal efficiency after amendments in Zone 2.

In Zone 2 (Figure 4), the lead removal efficiency averaged approximately 73%, with notable enhancements in samples containing a 15% wt. biochar concentration. This finding is consistent with previous studies [39] that highlight the effectiveness of biochar in stabilizing heavy metals in contaminated soils. Regarding biochar distribution, better results were observed when biochar was uniformly mixed with the contaminated soil. This is due to the fact that a uniform distribution of biochar in the soil would facilitate greater interaction between the biochar and the contaminants present, likely leading to enhanced effectiveness in contaminant removal. Previous research has shown that a uniform biochar distribution in soil can enhance its effectiveness in retaining contaminants and promoting beneficial microbial activity for soil remediation [40,41].

In this research, biochar application significantly improved the uptake of heavy metals in both zones, contributing to effective soil remediation. This is consistent with findings from Anne et al. [42], who reported that biochar derived from various bio-substrates, such as rapeseed and digestate, enhanced heavy metal accumulation in plants like buckwheat and white mustard. They found that rapeseed biochar was particularly effective in reducing the heavy metal content in soil and plant biomass, indicating its potential for phytostabilization and phytoextraction. Similarly, previous research by Sun et al. [43] demonstrated that biochar amendments improved the phytoremediation efficiency of crops like sunflower and maize in heavy-metal-contaminated soils. Their study highlighted that biochar not only enhances heavy metal uptake by plants but also improves soil properties, such as pH and nutrient availability. Ahmad et al. [44] showed that the use of biochar could achieve substantial Pb removal efficiencies, with some experiments reporting over a 65% reduction in Pb concentration in soils. Wang et al. [45] reported a lead (Pb) removal rate of 99.34% in solution and a decrease in lead bioavailability of 37.0% in soil treated with biochar, exceeding the 70% removal rate. A study of Hamzah et al. [46] found that plants treated with humic acid-coated biochar achieved Pb removal rates ranging from 40.04% to 87.28%, surpassing the 70% mark in some cases, as reported in the study.

### 3.4. Effects of Pb on Seed Germination and Seedling Growth

The seeds of *Lolium perenne* did not germinate in any of the contaminated soils or in the control soil. Although the Pb levels in Zone 1 were not excessively high enough to inhibit the growth of this species, factors such as the basic pH were decisive in its growth [37]. The seeds of *Brassica rapa* germinated in 100% of the amended soils in Zone 1, while in the second culture, no sprouts appeared in the samples with 5% wt. biochar. In both cultivations, the first stems appeared on day 6.

In Zone 2, the high lead levels inhibited plant growth during the first cultivation. During the second culture, some seeds began to germinate in the samples where biochar was mixed into the soil at an amount of 15% wt., but this growth was minimal. This delayed germination can be attributed to the biochar’s gradual remediation effects, which slowly improved soil conditions by reducing lead bioavailability and enhancing nutrient uptake, thereby eventually allowing for seed germination and plant growth [47]. The inhibitory effects of high lead concentrations on plant growth are well-established, as lead disrupts essential physiological processes such as photosynthesis and nutrient absorption [48]. The initial absence of growth followed by delayed germination aligns with biochar’s gradual improvement of soil properties, supporting its effectiveness in long-term soil remediation strategies. However, the persistently high lead levels, despite biochar application, underscore the need for extended remediation strategies to achieve a significant reduction.

*Brassica rapa* grew in 100% of the amended soils in Zone 1 and the uncontaminated soils. However, the percentage of germinated seeds and the length of the stem and roots varied considerable among the different soils. The uncontaminated soil produced plants with the longest stems and roots. It can be observed in Table 4 that the germination speed increased when the biochar was mixed with the soil, and the amount of 10% wt. biochar had a positive effect, obtaining the best results. Stem length was generally unaffected by the amount or distribution of biochar. A 10% wt. biochar treatment led to improvements in both zones. Previous studies have reported enhanced plant growth metrics, including stem length, in soils amended with biochar at similar concentrations [49]. Root length was also not affected in the soil mixtures with biochar; however, the amount of biochar had a significant effect on the contaminated soil with superficial biochar. Laird et al. [50] and Waters et al. [51] reported that biochar amendments often lead to better overall plant health and growth, although the impact on specific growth parameters like stem length can vary depending on the concentration and distribution of biochar in the soil. In Zone 2, it was not possible to determine these parameters due to the absence of growth or minimal growth, with only a small number of sprouts reaching a length of 1 cm.

**Figure 5:**
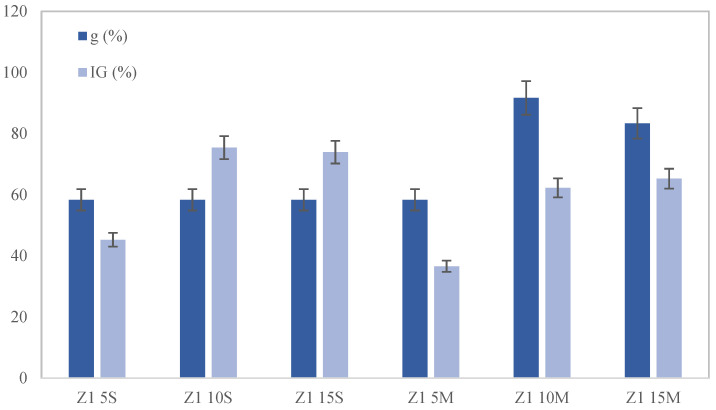
Effect of amendments on the germination index (IG) and germination coefficient (g).

The experimental results (Figure 5) indicate that seed germination was more successful in the samples where biochar was mixed into the soil rather than applied superficially. Among the various biochar treatments, the best outcomes were observed with a 10% wt. biochar amendment. This was evidenced by the highest germination rates and the fastest germination speed in the samples containing 10% wt. mixed biochar. These findings can be attributed to the enhanced soil properties provided by the mixed biochar. Numerous studies [18,52] have demonstrated that biochar incorporated into soil improves the soil’s structure, water retention, and nutrient availability more effectively than when it is merely spread on the surface. This creates a more favorable environment for seed germination and early plant development. Other studies [16,53] have demonstrated that mixing biochar into soil increases its porosity and water-holding capacity, thereby creating optimal conditions for seed germination and root growth. Biochar mixed into the soil also enhances microbial activity and nutrient cycling, which further supports plant health and growth [41,54].

The optimal 10% wt. biochar amendment likely provides the best balance between these benefits without overwhelming the soil ecosystem. Higher concentrations of biochar can sometimes lead to reduced soil fertility due to excessive carbon content, which can immobilize nutrients [55]. Conversely, lower concentrations might not provide sufficient improvement in soil properties to significantly affect germination and growth.

### 3.5. Effects of Pb Levels on Physiological Parameters

**Figure 6:**
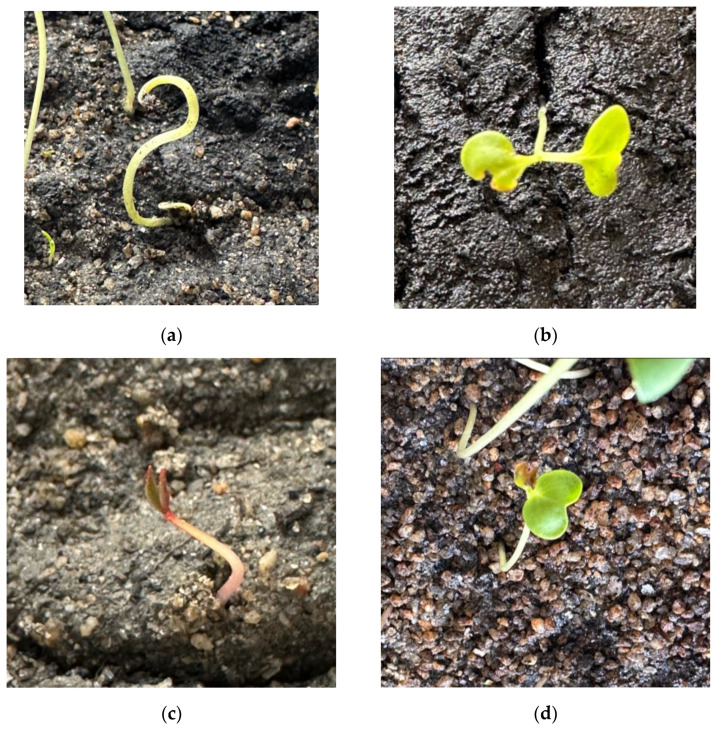
Effect of Pb on physiological parameters: (**a**) leafless plants, (**b**) yellow leaves, (**c**) chlorosis, and (**d**) wilted leaves.

During plant growth, several developmental anomalies were observed, predominantly attributed to the absorption of heavy metals through the roots. Examples include (i) plants exhibiting growth with absent leaves, where only the stem has developed (Figure 6a), (ii) leaves displaying yellowish coloration with corroded areas (Figure 6b), (iii) the development of small-sized, reddish plants (chlorosis) that fail to attain an average length (Figure 6c), and (iv) instances of leaves not reaching their expected size and becoming wilted (Figure 6d). These findings substantiate that the presence of lead in soils induces plant growth inhibition and disrupts photosynthetic processes. Islam et al. [48] examined the effect of lead on the photosynthesis and leaf ultrastructure of plant species with different ecological strategies, finding that the presence of lead in soil negatively affects the photosynthesis and leaf structure of plants. Giannakoula et al. [56] obtained similar results in *Citrus aurantium* plants.

### 3.6. Effectiveness of Amendments

A two-factor analysis of variance (ANOVA) was conducted to reflect the impact of the amount and distribution of biochar on lead removal from soil. The analysis was performed with an alpha value of 0.05. The results (Table 5) suggest that biochar distribution has a statistically significant effect on lead removal from the soil (*p* < 0.01), whereas the amount of biochar has no significant effect in either zone, with a *p* value higher than 0.19. This can be attributed to the high adsorption capacity of biochar, allowing heavy metals like lead to adhere to its surface. This adsorption property is influenced by how biochar is distributed in the soil. When biochar is evenly mixed into the soil, it maximizes the contact between the biochar and lead particles, enhancing adsorption efficiency. Studies have shown that incorporating biochar into the soil can significantly improve the immobilization of heavy metals, reducing their mobility and bioavailability [15,19,57]. Additionally, the surface application of biochar creates a barrier that can intercept heavy metals present in the topsoil layer, preventing them from leaching into deeper layers and entering the plant–root system [58]. This not only aids in lead removal but also prevents its uptake by plants, reducing toxicity [20]. Theorem-type environments (including propositions, lemmas, corollaries, etc.) can be formatted as follows:

The ANOVA analysis (Table 6) reveals the statistical significance of biochar distribution on germination rates (*p* < 0.05) and root length (*p* < 0.05). The 10% wt. biochar concentration yields the best results. These findings highlight the statistical significance of biochar distribution in influencing plant germination and growth. Numerous studies have investigated the effects of biochar on plant growth, often employing ANOVA to analyze the data and assess statistical significance. For instance, research by Jeffery et al. [59] explored the impact of biochar distribution on soil properties and plant growth. They observed that the spatial arrangement of biochar within the soil had notable effects on nutrient availability and root development, indicating the significance of biochar distribution in plant performance. Furthermore, an analysis conducted by Lehmann and Stephen [16] synthesized findings from various studies and concluded that biochar application positively influenced plant growth across different environments. On the other hand, the amount of biochar did not have a statistically significant effect on the seedling growth.

## 4. Conclusions and Perspectives for Future Studies

This study highlights the significant impact of biochar on the remediation of lead-contaminated soils. Moreover, the combined application of biochar and phytoremediation techniques has shown promising results. The results indicate that seed germination and plant development were notably superior in soil samples where biochar was mixed rather than applied superficially, with the optimal results observed at a 10% wt. biochar amendment. When biochar is mixed into the soil, its contact with lead particles is maximized, significantly enhancing adsorption efficiency and reducing the mobility and bioavailability of lead. In addition, it should be noted that the contact time between the biochar and the soil allows for an increased amount of lead removal.

This study showed that integrating biochar with phytoremediation can lead to improved growth rates and higher metal uptake in shooting range soils, making it a promising strategy for sustainable soil management and remediation. However, there are certain limitations that need to be addressed. Firstly, the study’s scope was limited to a specific type of biochar and plant species, which may not fully represent the wide variety of potential biochar–plant combinations available for remediation purposes. Future research should explore a broader range of biochar types derived from different feedstocks and diverse plant species to identify the most effective combinations for various soil conditions and contaminants.

Additionally, the long-term effects of biochar on soil health and heavy metal dynamics were not thoroughly examined. Longitudinal studies are necessary to understand the sustainability and potential cumulative impacts of biochar applications over extended periods of time. The interaction between biochar and soil microbial communities, which play an important role in bioremediation, also warrants further investigation.

Finally, our study primarily focused on the laboratory scale, and field trials are essential to validate these findings under real-world conditions. Environmental variables such as climate, soil type, and contamination levels can significantly influence the outcomes of biochar-assisted phytoremediation. Thus, large-scale field studies will help in assessing the practical applicability and scalability of this remediation approach.

## Figures and Tables

**Table 1:** Characteristics of the used biochar.

Parameter	Unit	Value
pH		7.6
Density	g/cm^3^	0.4
Particle size	mm	0.05–4
Carbon	%	67.1
Nitrogen	%	3.7
Hydrogen	%	9.2
Pb	mg/kg	2.7
S	mg/kg	4287
Specific area (BET)	m^2^/g	140
Water holding capacity	%	108

**Table 2:** Nomenclature of soil samples.

Ref.	Location	Biochar (% wt.)	Distribution
Z1 0	Zone 1	0	-
Z1 5S	Zone 1	5	superficial
Z1 10S	Zone 1	10	superficial
Z1 15S	Zone 1	15	superficial
Z1 5M	Zone 1	5	mixed
Z1 10M	Zone 1	10	mixed
Z1 15M	Zone 1	15	mixed
Z2 0	Zone 2	0	-
Z2 5S	Zone 2	5	superficial
Z2 10S	Zone 2	10	superficial
Z2 15S	Zone 2	15	superficial
Z2 5M	Zone 2	5	mixed
Z2 10M	Zone 2	10	mixed
Z2 15M	Zone 2	15	mixed

**Table 3:** Soil characterization.

	Uncontaminated Soil	Zone 1	Zone 2
Particle size distribution			
Sand (%)	99.8	99.9	99.6
Silt (%)	0.2	0.1	0.4
Soil pH	5.5	8.2	8.1
Electrical conductivity (μS/cm)	110	50	120
Pb (mg/kg)	46.2	171.0	116,486.0

**Table 4:** Soil characterization.

Ref.	Biochar (% wt.)	Distribution	Stem Length	Root Length	GS (S/d)
Z1 5S	5	superficial	5.4 ± 1.7	1.7 ± 0.9	0.88
Z1 10S	10	superficial	6.7 ± 0.8	2.9 ± 1.3	1.43
Z1 15S	15	superficial	6.3 ± 2.1	3.3 ± 0.7	0.87
Z1 5M	5	mixed	3.8 ± 1.0	1.6 ± 0.6	1.67
Z1 10M	10	mixed	6.0 ± 0.7	1.8 ± 1.3	2.45
Z1 15M	15	mixed	4.1 ± 2.1	1.7 ± 1.1	1.50

**Table 5:** Soil characterization.

	Variables	F-Value	*p*-Value
Zone 1	Biochar distribution	193.7	0.0006
	% Biochar	2.8	0.1905
Zone 2	Biochar distribution	83.6	0.0021
	% Biochar	1.4	0.3154

**Table 6:** Soil characterization.

	Variables	F-Value	*p*-Value
G	Biochar distribution	5.8	0.0914
	% Biochar	0.1	0.7888
g	Biochar distribution	2.5686	0.2294
	% Biochar	2.8823	0.1881
IG	Biochar distribution	54.3	0.004
	% Biochar	7.7	0.0697
GS	Biochar distribution	3.1	0.1902
	% Biochar	7.8	0.0685
RSLTI	Biochar distribution	10.2	0.0441
	% Biochar	8.4	0.0625
SLSTI	Biochar distribution	1.1	0.4687
	% Biochar	4.8	0.1171

## Data Availability

The original contributions presented in the study are included in the article, further inquiries can be directed to the corresponding author/s.
